# Complex formation of anti‐VEGF‐C with VEGF‐C released during blood coagulation resulted in an artifact in its serum pharmacokinetics

**DOI:** 10.1002/prp2.573

**Published:** 2020-03-03

**Authors:** Daniela Bumbaca Yadav, Arthur E. Reyes, Priyanka Gupta, Jean‐Michel Vernes, Y. Gloria Meng, Michelle G. Schweiger, Shannon L. Stainton, Germaine Fuh, Paul J. Fielder, Amrita V. Kamath, Ben‐Quan Shen

**Affiliations:** ^1^ Preclinical and Translational Pharmacokinetics and Pharmacodynamics Department Genentech, Inc. South San Francisco CA USA; ^2^ Biochemical and Cellular Pharmacology Department Genentech, Inc. South San Francisco CA USA; ^3^ Safety Assessment Department Genentech, Inc. South San Francisco CA USA; ^4^ Antibody Engineering Department Genentech, Inc. South San Francisco CA USA

**Keywords:** immunocomplex, matrix effects, pharmacokinetics, VEGF‐C

## Abstract

A phage‐derived human monoclonal antibody against VEGF‐C was developed as a potential anti‐tumor therapeutic and exhibited fast clearance in preclinical species, with notably faster clearance in serum than in plasma. The purpose of this work was to understand the factors contributing to its fast clearance. In vitro incubations in animal and human blood, plasma, and serum were conducted with radiolabeled anti‐VEGF‐C to determine potential protein and cell‐based interactions with the antibody as well as any matrix‐dependent recovery dependent upon the matrix. A tissue distribution study was conducted in mice with and without heparin infusion in order to identify a tissue sink and determine whether heparin could affect antibody recovery from serum and/or plasma. Incubation of radiolabeled anti‐VEGF‐C in human and animal blood, plasma, or serum revealed that the antibody formed a complex with an endogenous protein, likely VEGF‐C. This complex was trapped within the blood clot during serum preparation from blood, but not within the blood cell pellet during plasma preparation. Low level heparin infusion in mice slowed down clot formation during serum preparation and allowed for better recovery of the radiolabeled antibody in serum. No tissue sink was found in mice. Thus, during this characterization, we determined that the blood sampling matrix greatly impacted the amount of antibody recovered in the samples, therefore, altering its derived pharmacokinetic parameters. Target biology should be considered when selecting appropriate sampling matrices for PK analysis.

**Abbreviations**
BV: baculovirus
ELISA: enzyme‐linked immunosorbent assay
IACUC: Institutional Animal Care and Use Committee
IV: intravenous
NCA: non‐compartmental analysis
PBS: phosphate buffered solution
PK: pharmacokinetics
PT: prothrombin time
SE‐HPLC: size‐exclusion high‐performance liquid chromatography
VEGF‐C: vascular growth factor C

## 1 INTRODUCTION

Vascular endothelial growth factor‐C (VEGF‐C) is a soluble member of the VEGF family of ligands, also containing VEGF‐A, VEGF‐B, VEGF‐D, VEGF‐E, and placental growth factor.^1^ The structures among the members are similar with all of them containing the highly conserved VEGF homology domain.^2^ VEGF‐C is initially synthesized as a pre‐propeptide that undergoes both intracellular and extracellular processing before achieving its mature form as a homodimer that is held together through non‐covalent bonds at a molecular weight ~ 42 kDa.^2^, ^3^ It can interact with VEGF receptor‐2 (R2) and VEGFR‐3 to stimulate proliferation and migration of endothelial cells and increases vascular permeability.^3^ VEGF‐C mediates lymphangiogenesis via VEGFR‐3 and via a similar angiogenesis signally pathway as VEGF‐A via VEGFR‐2.^4^

VEGF‐C has wide expression in normal tissues, with measurable mRNA levels in muscle, thyroid, ovary, colon, liver, placenta, and spleen^1^ and measurable protein levels by immunohistochemistry in breast and prostate.^5^ It has also been shown to be expressed in various tumor tissues as well, such as thyroid, prostate, gastric, colorectal, lung, and breast.^6^ VEGF‐C increases tumor metastasis^7^ and by decreasing VEGF‐C expression in mammary tumors in mice, it was possible to decrease spontaneous metastasis and improve survival.^6^ Thus, VEGF‐C appears to be an important tumor angiogenic target.

An antibody was developed against VEGF‐C and in vivo studies were conducted in order to characterize its pharmacokinetics (PK) across several preclinical species including mouse, rat, and cynomolgus monkey. In all species tested, the anti‐VEGF‐C antibody cleared faster than a typical IgG1.^8^ Additionally, in mouse and rat, it had different PK properties depending on whether plasma or serum was collected. The goal of this work was to investigate the factors contributing to this fast clearance and understand the impact of matrix on PK. In vitro studies were performed to determine whether the antibody binds to blood cells, aggregates, degrades, or forms a complex with an endogenous protein. These studies were followed by mouse studies to verify whether the findings in vitro translated to in vivo. Furthermore, a tissue distribution study was conducted to determine whether the antibody distributed specifically to any tissues, which could also affect its in vivo clearance.

## 2 EXPERIMENTAL

All animal studies were conducted using protocols approved by each facility's Institutional Animal Care and Use Committee (IACUC).

### 2.1 PK study in athymic nude mice

In all, 18 experimentally naïve athymic nude mice (Charles River Laboratories, Hollister, CA) were administered intravenously anti‐VEGF‐C either at 1 mg/kg (n = 9) or 10 mg/kg (n = 9). Blood was collected from these mice periodically over 28 days and processed for either plasma (n = 6/group) or serum (n = 3/group). The plasma and serum samples were analyzed for anti‐VEGF‐C concentration using an antigen coat ELISA.

### 2.2 PK study in Sprague Dawley rats

In all, 18 experimentally naïve Sprague Dawley rats (Charles River Laboratories, Hollister, CA) were administered intravenously anti‐VEGF‐C either at 2 mg/kg (n = 6), 10mg/kg (n = 6), or 50 mg/kg (n = 6). Blood was collected from these rats periodically over 21 days and processed for either plasma (n = 3/group) or serum (n = 3/group). The plasma and serum samples were analyzed for anti‐VEGF‐C concentration using an antigen‐coated ELISA.

### 2.3 PK study in cynomolgus monkeys

In all, 16 experimentally naïve cynomolgus monkeys (Charles River Laboratories, Sparks, NV) were administered intravenously anti‐VEGF‐C either at 0.5 mg/kg (n = 4), 2 mg/kg (n = 4), 10 mg/kg (n = 4), or 50 mg/kg (n = 4). Blood was collected from these cynomolgus monkeys periodically over 56 days and processed for either plasma or serum. The plasma and serum samples were analyzed for anti‐VEGF‐C concentration using an antigen‐coated ELISA.

### 2.4 Antibody Iodination

Anti‐VEGF‐C was iodinated using an indirect iodogen addition method as previously described.^9^ The radiolabeled proteins were then purified using NAP5^TM^ columns (GE Healthcare Life Sciences) pre‐equilibrated in PBS. Following iodination, the iodinated material was characterized by size‐exclusion high‐performance liquid chromatography and ELISA as intact and retaining binding affinity for the target, respectively. The specific activity of the antibody was 8.12 µCi/µg.

### 2.5 In vitro characterization of anti‐VEGF‐C in whole blood, plasma, and serum

Female CD‐1 mouse (Charles River Laboratories) whole blood was collected into ethylenediaminetetraacetic acid (EDTA)‐coated blood collection tubes (BD, Franklin Lakes, NJ). Radioiodinated anti‐VEGF‐C was introduced into the blood along with (A) increasing amounts of unlabeled anti‐VEGF‐C at 0, 0.1, 0.5, 1, 10, and 100 µg/mL or (B) increasing amounts of recombinant human VEGF‐C (generated in‐house) at molar ratios of antigen to antibody of 0:1, 0.1:1, 0.5:1, 1:1, 5:1, and 10:1. The samples were gently mixed by inverting a few times and divided into three 250 µL aliquots. The aliquots were incubated at 4°C or 37°C with gentle rotation for 30, 90, or 180 minutes. After the incubation, the samples centrifuged to separate the plasma from the cell pellet and the plasma was analyzed by size‐exclusion HPLC (see method below). Some incubations were repeated with direct addition of the radioiodinated antibody into the mouse plasma or serum. PBS with 0.5% bovine serum albumin (BSA; Sigma‐Aldrich, St. Louis, MO) was used as a control matrix.

### 2.6 In vitro recovery of anti‐VEGF‐C from human and animal whole blood processed for plasma and serum

Radioiodinated anti‐VEGF‐C was introduced into CD‐1 mouse, human, cynomolgus monkey, and Sprague Dawley rat whole blood treated with EDTA (Bioreclamation LLC, Hicksville, NY for all species except mouse). The samples were gently mixed by inverting several times. Six 0.5 mL aliquots of blood were made and kept on ice for 30 minutes. Three of the six aliquots were centrifuged to separate the plasma samples from the cell pellets. The cell pellets were then washed with 0.5 mL cold PBS. Calcium chloride was added to the remaining three aliquots at a final concentration of 25 mmol/L. EDTA acts by absorbing the calcium required for proper blood clotting^10^ which is why calcium chloride was added to the blood to induce clot formation for serum preparation. The samples were immediately inverted and left at room temperature for 30 minutes until the blood had completely clotted. The samples were centrifuged to separate the serum from the clot. The clots were washed once with 0.5 mL cold PBS. The radioactivity in all samples was measured with a gamma counter (Wallac 1480 Wizard 3”, EC&G Wallac; Turku, Finland). Heparin and sodium citrate were also tested to determine whether antibody recovery was sensitive to the anti‐coagulant used, but no differences in recovery were noted (data not shown).

### 2.7 Anti‐VEGF‐C recovery from whole blood prepared from in vivo samples

All in vivo protocols, housing, and anesthesia were approved by the Institutional Animal Care and Use Committees of Genentech Laboratory Animal Resources, in compliance with the Association for Assessment and Accreditation of Laboratory Animal Care regulations. Female athymic nude mice obtained from Harlan Sprague Dawley (Hayward, CA) were used for the study. The mice were around 6‐ to 8‐weeks old. There were two study groups with 15 mice each. The mice received an IV bolus of either saline (control) or 50 IU/kg heparin followed by a constant infusion at 20 µL/h (1.25 IU/h) of either solution to maintain constant levels (based on previous internal unpublished data). The mice were infused for 30 minutes prior to dosing the study material and the infusion continued throughout the study. After 30 minutes, both groups received 600 µCi/kg [^125^I] anti‐VEGF‐C mixed with 10 mg/kg unlabeled anti‐VEGF‐C. Blood samples were collected from 3 mice in each group at 10 minutes and 1, 3, 5, and 8 hours post‐antibody dose. The blood samples were processed for both plasma and serum. The total radioactivity associated with each sample was measured in a gamma counter and total antibody concentrations were measure by ELISA (see below). The radioactivity is presented as the percent injected dose per milliliter of sample (%ID/mL).

### 2.8 Tissue distribution of anti‐VEGF‐C

Athymic nude mice (Harlan Sprague Dawley) received a single IV bolus of [^125^I]‐anti‐VEGF‐C at 400 µCi/kg (0.04 mg/kg) along with 0, 1, or 10 mg/kg unlabeled antibody. At 1, 24, and 96 hours post‐dose, blood was collected by cardiac puncture and processed for plasma using EDTA vacutainers. The following tissues were also harvest at each time point: liver, lungs, femur (for bone marrow), spleen, heart, stomach, whole small intestine, whole large intestine, mesenteric lymph nodes, kidneys, ovaries, and skeletal muscle (gastrocnemius). Each tissue was rinsed with PBS, blotted dry, and weighed. All samples were stored frozen at −70°C until analyzed for total radioactivity using a gamma counter. The radioactivity is presented as %ID/mL or %ID/g.

### 2.9 Size‐exclusion HPLC

Size‐exclusion HPLC was performed using a Phenomenex™ BioSep‐SEC‐S 4000, 300 × 7.8 mm, 5 µm column (No. 313834‐4, Phenomenex; Torreance, CA). The mobile phase was PBS, and the flow rate was 0.75 mL/min for 25 minutes. The ChemStation analog‐to‐digital converter (Agilent Technologies; Santa Clara, CA) was set to 25 000 units/mV, peak width 2 seconds, slit 4 nmol/L. [^125^I] was detected with a raytest Ramona 90 (raytest USA, Inc; Wilmington, NC) in line with a standard Agilent 1100 HPLC module system. The data were collected using ChemStation for LC 3D Systems (Revision B.01.03 [204], Agilent Technologies).

### 2.10 ELISA

The concentration of anti‐VEGF‐C in the plasma and serum samples at each time point were determined using an ELISA. Recombinant human VEGF‐C was used as the capture and goat anti‐human IgG Fc conjugated to horseradish peroxidase (HRP) was used for detection. The lower limit of quantitation was 0.234 ng/mL with a minimum dilution of 1:20.

### 2.11 Pharmacokinetic analysis

Concentration‐time data were used to estimate PK parameters with WinNonlin Enterprise, Version 5.1.1 (Pharsight Corporation). Non‐compartmental analysis (NCA) with sparse‐sampling (WinNonlin Model 201; IV Bolus input using uniform weighting and Linear Trapezoidal [Linear Interpolation] rule). For PK analysis in mice, a naïve pooled approach was used; hence, only a single estimate is provided for each PK parameter, and no standard deviation is reported.

## 3 RESULTS

### 3.1 Anti‐VEGF‐C PK in mice, rats, and cynomolgus monkeys

**Figure 1:**
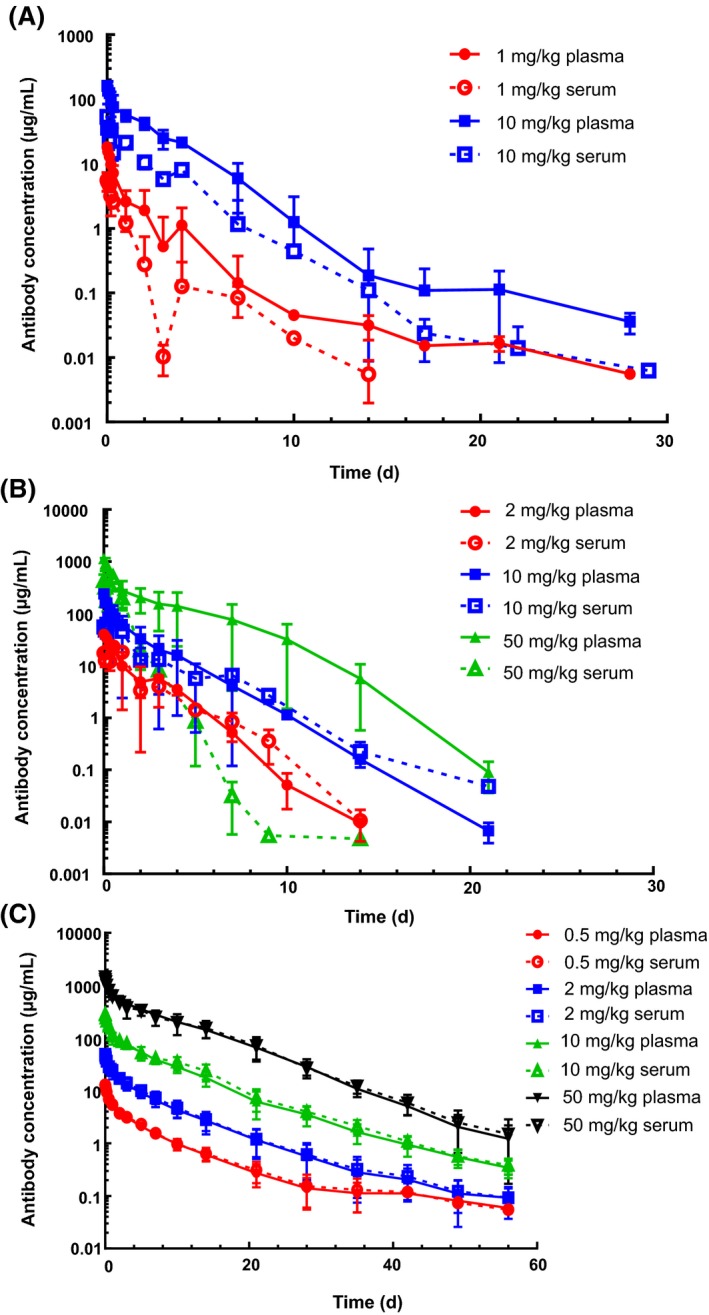
Pharmacokinetics in plasma and serum of the anti‐VEGF antibody in mice (A), rats (B), and cynomolgus monkeys (C)

Following single intravenous dose administration of anti‐VEGF‐C to mice, rats, and cynomolgus monkeys, the antibody displayed faster than expected clearance (Figure 1). In athymic nude mice, clearance in plasma decreased with increasing dose from 1 mg/kg to 10 mg/kg with values going from 78 mL/day/kg down to 38 mL/day/kg (Table 1). The clearance in serum was much faster, ranging from 225 mL/day/kg to 114 mL/day/kg at 1 and 10 mg/kg, respectively. Furthermore, the C_max_ in plasma was about three times higher than that in serum.

**Table 1:** Pharmacokinetic parameters of anti‐VEGF‐C in mouse plasma and serum

	Plasma		Serum	
	1 mg/kg	10 mg/kg	1 mg/kg	10 mg/kg
AUC (day*µg/mL)	13	255	4.0	70
CL (mL/day/kg)	78	38	225	114
Terminal T ½ (day)	2.2	2.0	1.3	1.8
Cmax (µg/mL)	18	164	5.8	57
Vss (mL/kg)	166	101	317	297

The trend in rats was different from that in mice with very slight decrease in plasma clearance on increasing dose and an opposite trend of an increase in serum clearance with dose (Table 2). The rat plasma clearance was 45, 46, and 36 mL/day/kg at 2, 10, and 50 mg/kg, respectively. Similar to mouse, clearance values in serum were higher than those in plasma, with 54, 99, and 120 mL/day/kg at 2, 10, and 50 mg/kg, respectively. Furthermore, the C_max_ in plasma was 1.5‐2.5 times higher than that in serum.

**Table 2:** Pharmacokinetic parameters of anti‐VEGF‐C in rat plasma and serum

	Plasma			Serum		
	2 mg/kg	10 mg/kg	50 mg/kg	2 mg/kg	10 mg/kg	50 mg/kg
AUC (day*µg/mL)	46 ± 5	233 ± 94	1670 ± 868	43 ± 1.4	147 ± 91	477 ± 65
CL (mL/day/kg)	45 ± 5	46 ± 23	36 ± 23	54 ± 1.8	99 ± 64	120 ± 12
Terminal T ½ (day)	1.0 ± 0.2	1.2 ± 0.5	1.1 ± 0.2	1.1 ± 0.1	1.2 ± 0.8	0.5 ± 0.2
Cmax (µg/mL)	40 ± 0.6	239 ± 17	1137 ± 158	24 ± 5.2	94 ± 51	549 ± 42
Vss (mL/kg)	76 ± 18	62 ± 15	73 ± 16	103 ± 18	155 ± 99	84 ± 9

Note

Values are mean ± standard deviation (n = 6).

In cynomolgus monkeys, yet a different trend was observed. There was a smaller decrease in clearance in both plasma and serum as the dose level increased, but unlike in mouse and rat, there was no difference in clearance values between the two matrices (Table 3). In both matrices, the clearance is about 13 mL/day/kg at 0.5 mg/kg and 8 mL/day/kg at 50 mg/kg. Furthermore, there was little difference in C_max_ between the matrices.

**Table 3:** Pharmacokinetic parameters of anti‐VEGF‐C in cynomolgus monkey plasma and serum

	Plasma				Serum			
	0.5 mg/kg	2 mg/kg	10 mg/kg	50 mg/kg	0.5 mg/kg	2 mg/kg	10 mg/kg	50 mg/kg
AUC (day*µg/mL)	37.3 ± 4.40	156 ± 39.0	869 ± 142	6090 ± 993	38.5 ± 5.44	169 ± 53.0	998 ± 112	5880 ± 1380
CL (mL/day/kg)	13.6 ± 1.56	13.5 ± 3.31	11.7 ± 1.68	8.36 ± 1.26	13.2 ± 1.75	12.7 ± 3.88	10.1 ± 1.10	8.81 ± 1.77
Terminal T ½ (day)	5.36 ± 1.92	5.19 ± 1.24	5.46 ± 0.843	6.23 ± 0.645	5.43 ± 1.74	5.36 ± 1.16	6.19 ± 0.749	6.95 ± 1.38
Cmax (µg/mL)	12.3 ± 1.59	45.4 ± 6.52	248 ± 24.3	1360 ± 170	12.7 ± 1.71	47.2 ± 6.25	280 ± 24.4	1380 ± 227
Vss (mL/kg)					89.9 ± 15.3	84.2 ± 10.4	79.8 ± 6.05	81.8 ± 23.4

Note

Values are mean ± standard deviation (n = 4).

### 3.2 In vitro characterization of anti‐VEGF‐C in whole blood, plasma, and serum

**Figure 2:**
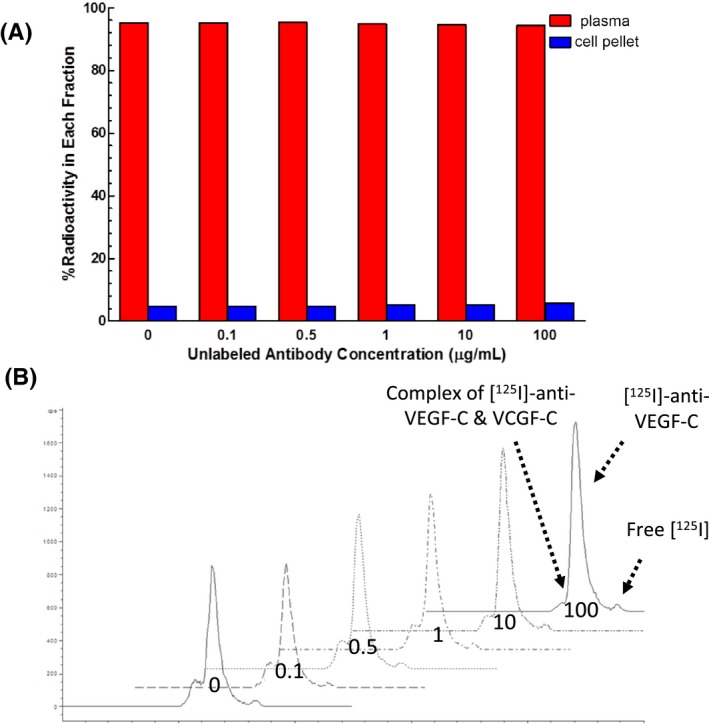
(A) In vitro recovery of [^125^I]‐anti‐VEGF‐C from mouse whole blood processed for plasma and cell pellet. (B) Size‐exclusion HPLC analysis of plasma samples processed from mouse whole blood incubated with [^125^I]‐anti‐VEGF‐C with increasing amounts of unlabeled antibody. The antibody concentrations increased from left to right: 0, 0.1, 0.5, 1, 10, and 100 μg/mL. The low molecular weight peak corresponded to free [^125^I] in the sample and is the right most peak, the main peak corresponded to the intact antibody and is the middle peak, and the high molecular weight peak corresponded to a protein complex and is the left most peak

In order to elucidate the cause of this faster than expected clearance in all species as well as the difference in clearance between plasma and serum in mice and rats, in vitro studies were conducted. Investigation to determine whether anti‐VEGF‐C bound to blood cells, aggregated, degraded, or formed a complex with an endogenous mouse protein was conducted to see if any of these reasons could mechanistically explain these findings. Radiolabeled anti‐VEGF‐C was incubated in mouse whole blood with increasing amounts of unlabeled antibody and then, the blood was separated into plasma and cell pellet fractions. A majority of the radioactivity was found in the plasma fraction of whole blood, with minimal amounts detected in the cell pellets at all concentrations of antibody (Figure 2A). The plasma was analyzed on the size‐exclusion HPLC and along with the [^125^I]‐anti‐VEGF‐C peak, corresponding to a retention time of 13 minutes, a high molecular weight peak was detected at a retention time of 11.5 minutes (Figure 2B). Some free [^125^I] was also detected in the samples at a retention time of 17 minutes. The high molecular weight peak decreased as the amount of unlabeled anti‐VEGF‐C increased, changing from about 14% total radioactivity when no unlabeled antibody was added down to about 3% total radioactivity in the presence of 100 µg/mL unlabeled anti‐VEGF‐C (Table 4). The reduction in magnitude of the high molecular weight peak suggests specific interaction of the antibody with an endogenous plasma protein(s).

**Table 4:** Percent of the area under the size‐exclusion HPLC peaks of plasma from an incubation of [^125^I]‐anti‐VEGF‐C in mouse whole blood along with increasing concentrations of unlabeled anti‐VEGF‐C

Unlabeled Antibody Concentration (µg/mL)	High Molecular Weight Peak	Main Peak	Low Molecular Weight Peak
0	14.2%	75.1%	10.7%
0.1	16.0%	75.1%	8.95%
0.5	14.0%	75.8%	10.2%
1	13.4%	76.6%	10.0%
10	6.53%	83.0%	10.4%
100	3.30%	85.0%	11.7%

In order to determine if the protein(s) associate with the high molecular weight peak is endogenous VEGF‐C, increasing amounts of recombinant human VEGF‐C was added to blood along with the radiolabeled antibody. As the molar ratio of VEGF‐C to antibody was increased, so did the intensity of the high molecular weight peak (Figure S1). Meanwhile, the peak intensity for [^125^I]‐anti‐VEGF‐C decreased. At a molar ratio of 0:1, the high molecular weight peak was ~6% total radioactivity, while at a molar ratio of 10:1, it was ~30% total radioactivity (Table 5). Furthermore, the high molecular weight peak had a retention time of 11.5 minutes, consistent with the retention time observed for the high molecular weight peak when no exogenous VEGF‐C was added.

**Table 5:** Percent of the area under the size‐exclusion HPLC peaks of plasma from an incubation of [^125^I]‐anti‐VEGF‐C in mouse whole blood along with increasing concentrations of unlabeled VEGF‐C

Molar Ratio VEGF‐C: anti‐VEGF‐C	High Molecular Weight Peak	Main Peak	Low Molecular Weight Peak
0:1	5.67%	92.3%	2.01%
0.1:1	5.55%	93.7%	0.776%
0.5:1	9.62%	88.1%	2.29%
1:1	12.4%	85.6%	2.04%
5:1	25.9%	73.1%	1.08%

When the antibody was added directly to plasma and serum, the high molecular weight peak was bigger in the serum sample than in the corresponding plasma sample (Figure S2). The serum high molecular weight peak was about 24% total radioactivity and the plasma high molecular weight peak was about 12% (Table S1). The addition of 100 µg/mL unlabeled anti‐VEGF‐C decreased the high molecular weight peaks in both matrices to about the same level.

### 3.3 In vitro recovery of anti‐VEGF‐C from human and animal whole blood processed for plasma and serum

**Figure 3:**
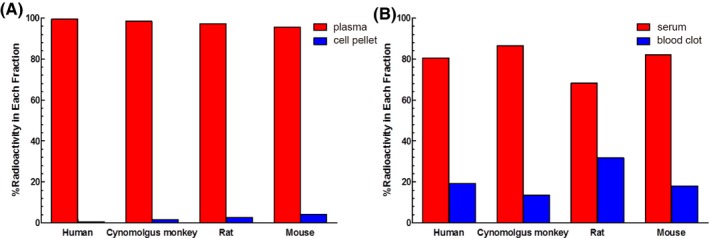
In vitro recovery of [^125^I]‐anti‐VEGF‐C from human, cynomolgus monkey, rat, and mouse whole blood processed either for plasma (A) or serum (B). The percent of radioactivity associated with the plasma and cell pellets or serum and blood clots are graphed

When processed for plasma, most of the radioactivity recovered from the whole blood was in the plasma fraction in all species tested (Figure 3A). The recovery in plasma ranged from 95% in mouse plasma to 99% in human plasma. In the whole blood processed for serum and blood clot, a majority of the radioactivity was also detected in the serum fraction in all species, but was generally lower than what was detected in the plasma (Figure 3B). The recovery in serum ranged from 70% in rat serum to 85% in cynomolgus monkey serum. The blood clots had correspondingly higher radioactivity levels than the cell pellets.

### 3.4 Anti‐VEGF‐C recovery from whole blood prepared from in vivo samples

**Figure 4:**
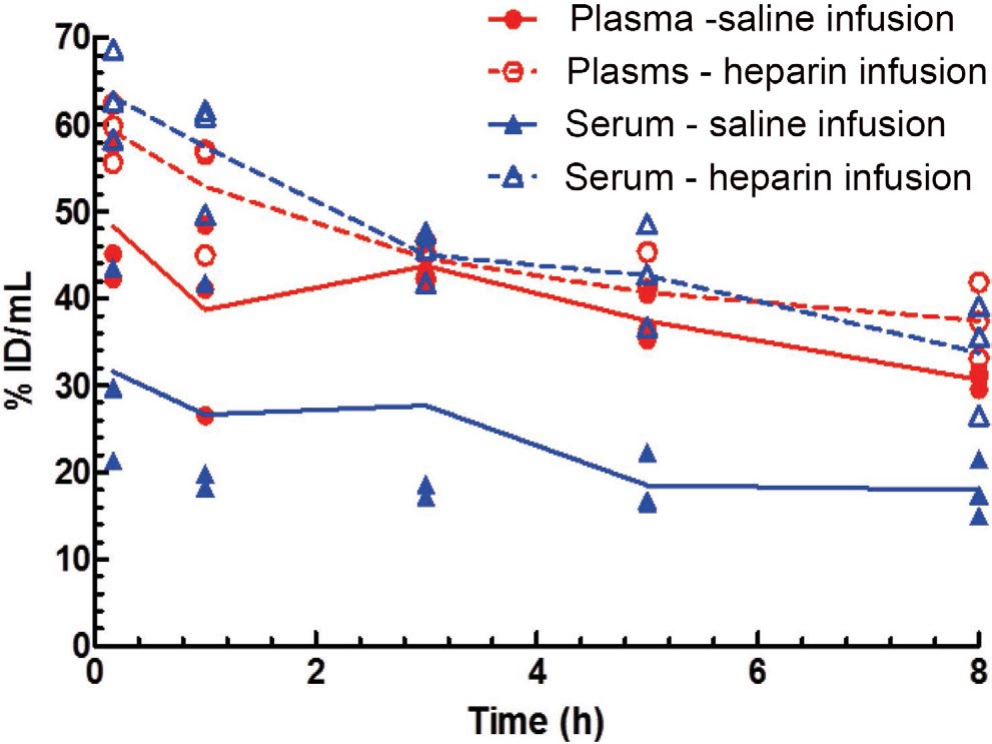
Plasma and serum concentrations determined by radioactivity level from mice administered [^125^I]‐anti‐VEGF‐C intravenously along with 10 mg/kg unlabeled antibody while being infused with either saline or heparin. The average μg‐Equivalents/mL concentrations ± standard deviation are graphed (n = 3)

In order to determine whether the in vitro phenomenon would also be observed when in vivo blood samples were prepared for plasma and serum, a short radioactive pharmacokinetic study was conducted. There was about 2‐fold higher [^125^I]‐anti‐VEGF‐C levels in the serum samples from the heparin infused mice than in those from the saline infused mice (Figure 4). There was a trend toward higher [^125^I]‐anti‐VEGF‐C levels in the plasma from the heparin infused mice than those from the saline infused mice, although this difference was not significant. In the saline infused mice, the plasma had about a 1.5‐2‐fold higher [^125^I]‐anti‐VEGF‐C levels than the serum at all time points. In the heparin infused mice, there was no significant difference between plasma and serum [^125^I]‐anti‐VEGF‐C levels.

The overall antibody exposure in plasma from both the saline and heparin infused mice along with that in serum from those infused with heparin were similar, with AUC_0‐0.33d_ of 38.3 ± 1.30 day*µg/mL, 44.2 ± 1.74 day*µg/mL, and 36.6 ± 2.02 day*µg/mL, respectively. The AUC_0‐0.33d_ in serum from animals infused with saline was lower at 9.42 ± 1.80 day*µg/mL. Furthermore, the estimated serum clearance for the saline infused group also trended faster at about 516 mL/day/kg compared with ~84.2 mL/day/kg for plasma from those infused with saline, ~94.3 mL/day/kg for plasma from those infused with heparin, and ~92.4 mL/day/kg for serum from those infused with heparin.

### 3.5 Tissue distribution of anti‐VEGF‐C

In order to determine whether the antibody distributed specific to any tissues as this could also contribute to fast clearance, a 4‐day mouse tissue distribution study was conducted. No tissue sink was identified in this study (Figure S3).

## 4 DISCUSSION

The goal of these studies was to understand what contributed to the fast clearance of anti‐VEGF‐C in mice, rats, and cynomolgus monkeys and why there were differences in clearance between plasma and serum in mice and rats. Nonlinear kinetics was observed in mice between 1 and 10 mg/kg, with clearance decreasing as dose was increased and faster clearance estimated in serum compared to plasma. The nonlinearity in clearance over the dose range of 2‐50 mg/kg was less apparent in rat plasma, but recapitulated in rat serum with the surprisingly opposite trend of clearance increasing with increase in dose. In general, the clearance estimates in rat serum were also higher than those in rat plasma. Slight non‐linearity was observed in cynomolgus monkey plasma and serum over the dose range of 0.5‐50 mg/kg with clearance decreasing as dose was increased and different from mouse and rat in terms of seeing no difference in cynomolgus monkeys suggests that target‐mediated clearance mechanisms could be dominating elimination of the antibody and this can be saturated with higher doses. However, this explanation would not hold for rat serum since there was an increase in clearance with increase in dose and the exact mechanism responsible for this trend warrants further investigation.

Another unexpected finding was the matrix difference in clearance estimates between rodent plasma and serum, but not in cynomolgus monkey with unknown mechanism. Nonspecific binding interactions for this antibody were assessed in the baculovirus (BV) assay.^8^ The antibody had a normalized binding score of 0.4, which is below the assay cutoff of 1 for increased risk of nonspecific interactions. The impact of charge on nonspecific interactions was also examined using an in silico charge assessment tool.^11^ This anti‐VEGF‐C antibody has an Fv charge of 1.4, which is within the hypothesized low risk range for nonspecific binding. As the current screening assays for atypical PK suggest that the antibody is at low risk for fast clearance due to nonspecific interactions, we considered whether the antibody PK could be affected by other mechanisms.

A tissue distribution study was conducted in mice to rule out the possibility of specific distribution of the antibody to a tissue sink as contributing the fast clearance of this antibody. A number of tissues were evaluated, including liver, spleen, heart, kidneys, lungs, among others and no tissue sink was identified (Figure S3).

In order to understand the factors contributing to the fast clearance in rodents, the stability of anti‐VEGF‐C in whole blood was determined by incubating [^125^I]‐anti‐VEGF‐C along with increasing amounts of unlabeled antibody in mouse whole blood, processing it for plasma, and analyzing the plasma by size‐exclusion HPLC. The majority of the radioactivity was detected in the plasma fraction of whole blood (Figure 2A). The radiolabeled antibody formed a high molecular weight complex in mouse plasma that was competed with excess amounts of unlabeled antibody, signifying that the formation of high molecular weight species was due to specific, saturable binding (Figure 2B and Table 4). The high molecular weight complex was approximately 5‐fold larger than the antibody, based on its retention time on the size‐exclusion HPLC and could be due to anti‐VEGF‐C aggregation or complex formation with an endogenous protein.

Given that anti‐VEGF‐C targets a soluble protein, the high molecular weight peak could be a complex formed between the antibody and its target. In order to test whether VEGF‐C was involved in high molecular weight peak formation, increasing molar ratios of recombinant human VEGF‐C were added to mouse blood containing [^125^I]‐anti‐VEGF‐C. There was a clear increase in the magnitude of the high molecular weight peak in plasma as the molar ratio of VEGF‐C to anti‐VEGF‐C increased, with no shift in retention time (Table 5 and Figure S1). These data suggest that the antibody was forming a complex with VEGF‐C, rather than the antibody aggregating.

Plasma and serum contain significant differences in the level of certain analytes.^12^, ^13^ Consistent with human serum having ~2x higher endogenous VEGF‐C levels due to its enhanced release during blood coagulation as a result of platelet activation,^14^, ^15^, ^16^ the high molecular weight peak was larger in magnitude in serum than in plasma (Figure S2). After noticing a difference in the magnitude of the high molecular weight peak between the plasma and serum, recovery from human and animal whole blood processed for either of these matrices was assessed. Across all species tested, plasma recovered more radioactivity than serum (Figure 3), although minor species differences in recovery from serum were noted (rat having the lowest recovery and cynomolgus monkeys having the highest). The antibody cross‐reacts with VEGF‐C from rat and human with a K_D_ of about 1 nmol/L (Table S2) and the VEGF‐C sequence is conserved across species,^17^ so it is unlikely that the difference in recovery among the species is due to difference in the antibody's ability to bind the antigen. Furthermore, the blood clotting time as assessed by the prothrombin time blood test (PT) is fairly comparable for all species^18^, ^19^ and is not likely to be the reason for these differences. The species differences in recovery may be due to variable levels of VEGF‐C release upon platelet activation. The balance of the radiolabeled antibody that was not detected in the plasma or serum was found associated either with the cell pellet or the blood clot. The higher radioactive signal being associated with the blood clot is consistent with the hypothesis that the antibody is binding to VEGF‐C as it is released from platelets and being trapped within the blood clot as coagulation proceeds. This is also consistent with the observation from the PK studies in which lower exposures were observed in the serum of mice and rats compared to that in serum (Figure 1 **, **Table 1, and Table 2). Furthermore, there were lower C_max_ values in serum of these species compared to that in plasma, also suggesting that this phenomenon was an artifact of the different matrix preparations.

Interestingly, although we observed lower recovery in serum compared to plasma for the cynomolgus monkeys blood samples in vitro, there was no difference in PK between these two matrices (Figure 1 and Table 3). It is not clear as to why there is discordance between the cynomolgus monkeys in vitro and in vivo results. Perhaps adding excess amounts of calcium chloride to blood treated with EDTA resulted in more VEGF‐C to be released in the in vitro assay than is released during typical blood coagulation, and cynomolgus monkeys may be more sensitive to this than other species. One potential approach to understand the lack of in vitro to in vivo correlation for this species would be to test the recovery of anti‐VEGF‐C in untreated serum tubes (whole blood without EDTA treatment/spiking calcium chloride) though it is technically quite challenge to run such experiment in vitro. This discrepancy also highlights the challenges in translating information from in vitro to in vivo, and between animal species.

Based on these results, an in vivo study was conducted in mice to compare the antibody recovery in plasma and serum in order to test the hypothesis that the apparent difference in clearance between plasma and serum was due sample preparation. The plasma did have about 1.5‐2‐fold higher recovery of [^125^I]‐anti‐VEGF‐C than the serum (Figure 4), which translated into very different clearances (84.2 mL/day/kg vs. 516 mL/day/kg for plasma and serum, respectively) and exposures (38.3 µg/mL*day vs. 9.42 µg/mL*day for plasma and serum, respectively) for the two matrices. Since VEGF‐C is released from activated platelets during blood coagulation^16^ and heparin is known to slow clotting time of patients receiving low doses of unfractionated heparin,^20^, ^21^, ^22^, ^23^, ^24^, ^25^ it was hypothesized that maintaining the mice on a heparin infusion would also slow the blood clotting time and may allow for better recovery of the radiolabeled antibody from serum. Indeed, there was no significant difference between the plasma and serum radioactivity levels of the heparin infused mice (Figure 4), consistent with our hypothesis; the prolonged clotting time allowed for better recovery of the antibody in the serum. Taken together, these data suggest that anti‐VEGF‐C is trapped in the clot during serum preparation, lowering the detectable serum anti‐VEGF‐C levels and resulting in an apparent faster clearance than plasma.

Thus, through this characterization, we have demonstrated that the blood sampling matrix selection greatly impacted the amount of anti‐VEGF‐C antibody recovered in the samples due to the release of endogenous VEGF‐C resulted from blood clotting/platelet aggregation, therefore, altering its derived serum pharmacokinetic parameters. Though this is a case report, the finding may apply to other molecules that target to the antigens released into serum following platelet aggregation/degranulation such as VEGF‐A and several other growth factors. Therefore, our results clearly highlight that the biology of the target should be taken into account when selecting an appropriate matrix for PK analysis.

## DISCLOSURE

All authors were employees of Genentech, Inc, a member of the Roche Group, at the time the work was completed.

## AUTHORS’ CONTRIBUTION

Daniela Bumbaca Yadav contributed to the study design, experiment implementation, data analysis, and manuscript drafting; Arthur E. Reyes II contributed to cynomolgus monkey PK study and data analysis; Priyanka Gupta was responsible for rat and mice PK study; Jean‐Michel Vernes and Y. Gloria Meng were responsible for PK assay of anti‐VEGF‐C; Michelle G. Schweiger and Shannon L. Stainton were responsible for in vivo animal studies and tissue harvesting; Germaine Fuh contributed to the anti‐VEGF‐C antibody engineering; Paul Fielder contributed to study hypothesis and design; Amrita V. Kamath contributed to the PK study design, data analysis, and drafting manuscript; Ben‐Quan Shen contributed to the overall study design, data analysis, manuscript drafting, and final submission.

## Supporting information

FigS1

FigS2

FigS3

TableS1

TableS2
