# Supplementary figures and images for: Complex formation of anti‐VEGF‐C with VEGF‐C released during blood coagulation resulted in an artifact in its serum pharmacokinetics
Source: Pharmacol Res Perspect. 2020 Mar 3;8(2):e00573. doi: 10.1002/prp2.573 (PMC7053556; doi:10.1002/prp2.573)

Fig. S1

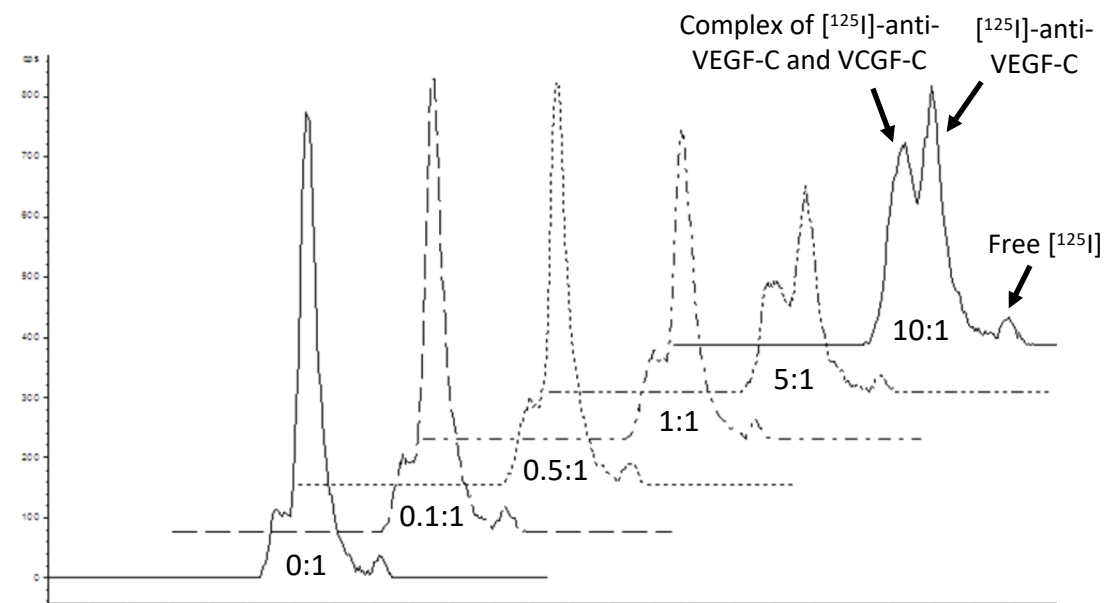

Supplement: Supplementary file 1 — FigS1 [file PRP2-8-e00573-s001.pdf]

Fig. S2

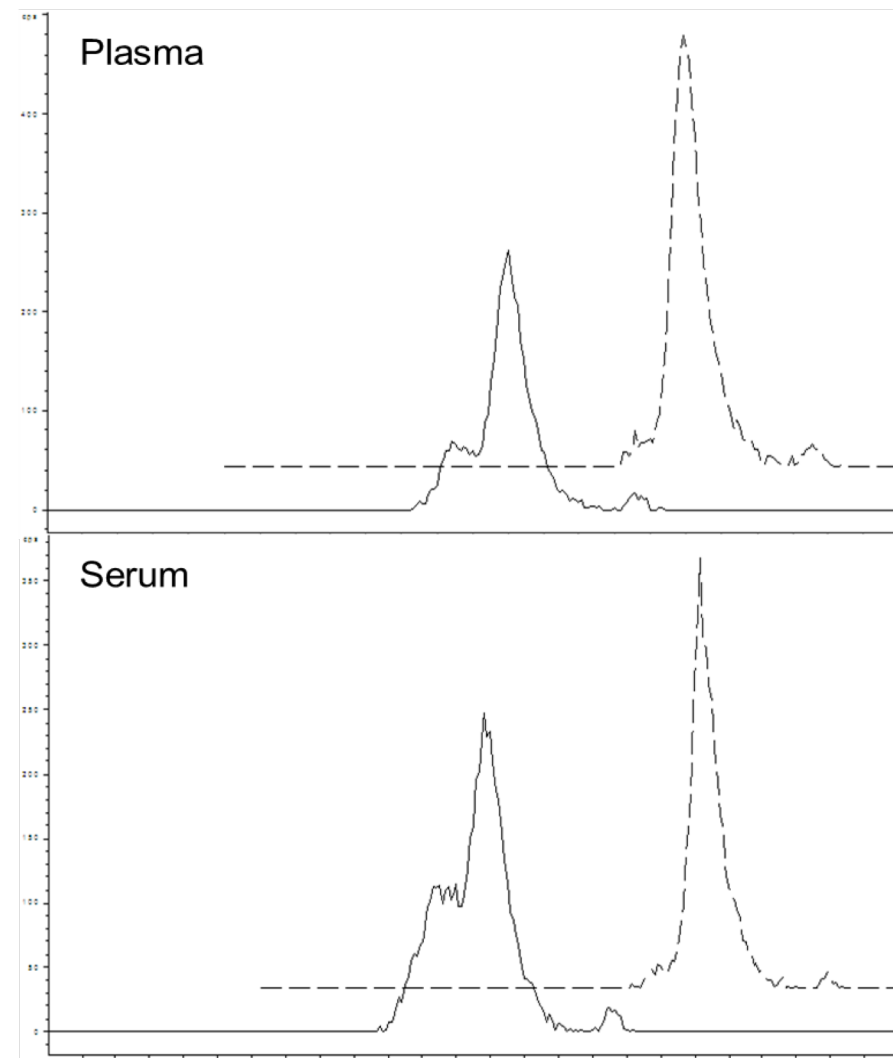

Supplement: Supplementary file 2 — FigS2 [file PRP2-8-e00573-s002.pdf]

Fig. S3

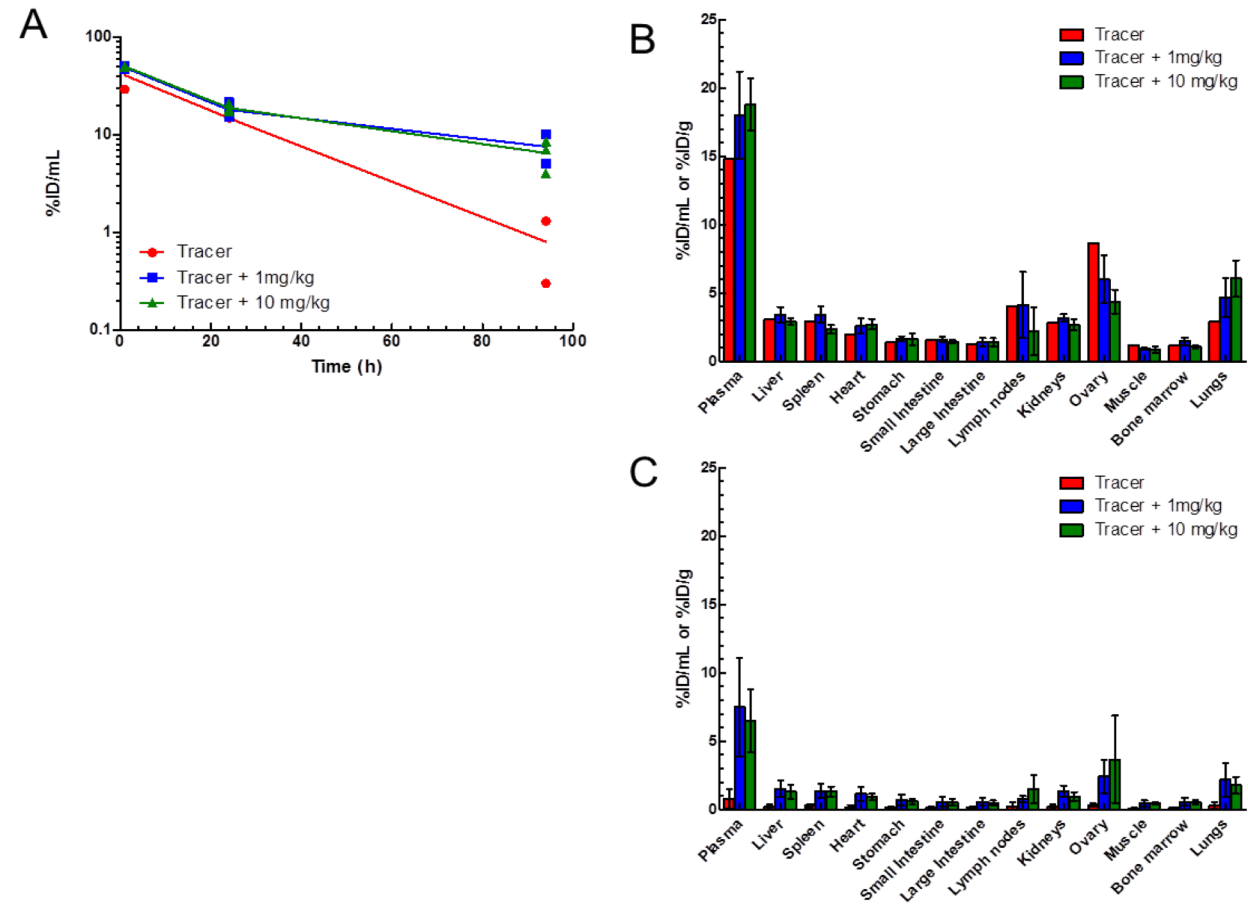

Supplement: Supplementary file 3 — FigS3 [file PRP2-8-e00573-s003.pdf]
