# Supplementary material for: Complex formation of anti‐VEGF‐C with VEGF‐C released during blood coagulation resulted in an artifact in its serum pharmacokinetics
Source: Pharmacol Res Perspect. 2020 Mar 3;8(2):e00573. doi: 10.1002/prp2.573 (PMC7053556; doi:10.1002/prp2.573)
Supplement: Supplementary file 4 — TableS1 [file PRP2-8-e00573-s004.docx]

**Table S1:** Percent of the area under the size-exclusion HPLC peaks of plasma and serum from an incubation [^125^I]-anti-VEGF-C ± unlabeled anti-VEGF-C in mouse whole blood.

| Matrix | Unlabeled Antibody Concentration (µg/mL) | High Molecular Weight Peak | Main Peak | Low Molecular Weight Peak |
| --- | --- | --- | --- | --- |
| Plasma | 0 | 12.4% | 86.2% | 1.43% |
|  | 100 | 1.90% | 97.1% | 1.03% |
| Serum | 0 | 24.1% | 74.8% | 1.12% |
|  | 100 | 1.42% | 98.2% | 0.379% |
