# Supplementary material for: Complex formation of anti‐VEGF‐C with VEGF‐C released during blood coagulation resulted in an artifact in its serum pharmacokinetics
Source: Pharmacol Res Perspect. 2020 Mar 3;8(2):e00573. doi: 10.1002/prp2.573 (PMC7053556; doi:10.1002/prp2.573)
Supplement: Supplementary file 5 — TableS2 [file PRP2-8-e00573-s005.docx]

**Table S2:** Binding kinetics and affinity of anti-VEGF-C to human and rat VEGF-C as determined by Surface Plasmon Resonance (SPR)

| Species | Kon (M^-1^*s^-1^) | Koff (s^-1^) | Kd (nM) |
| --- | --- | --- | --- |
| Human | 3.02 X 10^4^ | 3.10 X 10^-5^ | 1.03 |
| Rat | 3.37 X 10^4^ | 4.52 X 10^-5^ | 1.34 |
